# The role of INDY in metabolism, health and longevity

**DOI:** 10.3389/fgene.2015.00204

**Published:** 2015-06-09

**Authors:** Ryan P. Rogers, Blanka Rogina

**Affiliations:** ^1^Department of Sciences, Wentworth Institute of Technology, Boston, MA, USA; ^2^Department of Genetics and Genome Sciences, Institute for Systems Genomics, School of Medicine, University of Connecticut Health Center, Farmington, CT, USA

**Keywords:** aging, *Drosophila melanogaster*, *Indy*, caloric restriction, longevity genes

## Abstract

*Indy (I’m Not Dead Yet)* encodes the fly homolog of a mammalian SLC13A5 plasma membrane transporter. INDY is expressed in metabolically active tissues functioning as a transporter of Krebs cycle intermediates with the highest affinity for citrate. Decreased expression of the *Indy* gene extends longevity in *Drosophila* and *C. elegans*. Reduction of INDY or its respective homologs in *C. elegans* and mice induces metabolic and physiological changes similar to those observed in calorie restriction. It is thought that these physiological changes are due to altered levels of cytoplasmic citrate, which directly impacts Krebs cycle energy production as a result of shifts in substrate availability. Citrate cleavage is a key event during lipid and glucose metabolism; thus, reduction of citrate due to *Indy* reduction alters these processes. With regards to mammals, mice with reduced *Indy (mIndy^–/–^)* also exhibit changes in glucose metabolism, mitochondrial biogenesis and are protected from the negative effects of a high calorie diet. Together, these data support a role for *Indy* as a metabolic regulator, which suggests INDY as a therapeutic target for treatment of diet and age-related disorders such as Type II Diabetes and obesity.

## Introduction

The *Drosophila I’m Not Dead Yet (Indy)* gene encodes a plasma membrane transporter of Krebs cycle intermediates with highest affinity for citrate (Rogina et al., 2000; Knauf et al., 2002, 2006). In flies INDY is predominantly expressed in the midgut, which is important for food absorption; the fat body, which modules glycogen and fat storage, and oenocytes (fly liver), which is the site of lipid mobilization and storage (Rogina et al., 2000; Knauf et al., 2002; Frankel and Rogina, 2012; Rogers and Rogina, 2014).Thus, reduction in INDY reduces uptake, synthesis and storage of nutrients and affects metabolic activity. Reduction of *Indy* expression in both flies and worms extends longevity by a mechanism that is reminiscent of calorie restriction (CR), which is an environmental manipulation that extends longevity in a variety of species (McCay et al., 1935; Fei et al., 2003; Wang et al., 2009). Flies with reduced INDY levels experience many of the physiological changes that are commonly observed in CR flies. Such changes include altered lipid metabolism and insulin signaling, as well as enhanced mitochondrial biogenesis and spontaneous activity (Wang et al., 2009; Rogers and Rogina, 2014).

Studies investigating the function of mammalian *Indy* (*mIndy*) show the highest levels of expression in the liver and brain (Inoue et al., 2002). Similar to the trend of *Indy* expression in flies, mRNA levels were found to change during starvation in rat hepatocytes and mice liver. Furthermore, studies in *mIndy^–/–^* mice show similar effects in mitochondrial function, as well as lipid and glucose metabolism in the liver as those previously described in less complex organisms and in mice on CR (Fei et al., 2004; Wang et al., 2009; Birkenfeld et al., 2011). Together, these data suggest that the level and location of INDY serves to regulate and possibly mediate metabolic responses to nutrient availability during aging.

## The SLC13 Family of Transporters

INDY is a member of the SLC13 family of transporters in mammals, invertebrates, plants, and bacteria (Pajor, 2006, 2014). This class of transporters has variation in function with three members serving as sodium-coupled transporters for dicarboxylates/citrate (SLC13A2, SCL13A3, SLC13A5) and two members, which transport sulfates (SLC13A1 and SLC13A4). In mammals, SLC13A2 is mostly expressed on the apical membranes of the renal proximal tubule and small intestinal cells. Its primary function is to provide the energy required for normal cell function and balance of urinary citrate levels. SLC13A3 is expressed in a variety of tissues such as liver, brain, placenta, kidney, eye, and pancreas. SLC13A3 has a primary role in nutrient absorption, as well as drug and xenobiotic excretion. Finally, SLC13A5 (*mIndy*) has the highest sequence and functional similarity to *Drosophila Indy*. There is 34% identity and 50% similarity between the predicted INDY protein in flies and the human and rat sodium dicarboxylate transporter. In flies INDY is mainly expressed in the midgut, fat body and oenocytes (Rogina et al., 2000; Knauf et al., 2002; Frankel and Rogina, 2012; Rogers and Rogina, 2014). Similar to the metabolic tissue expression patterns found in fly tissue, the worm homolog of INDY is most robustly expressed throughout the intestinal tract (Fei et al., 2004). In mammals, INDY is predominantly expressed in the liver, testis, and brain, although expression is also found in the testis, placenta and kidneys (Yodoya et al., 2006; Pajor, 2014).

Physiological studies in Xenopus oocytes indicate that fly INDY is a plasma membrane exchanger for Krebs cycle intermediate with highest affinity for citrate, but can also transport succinate, oxaloacetate, fumarate, and α-ketoglutarate (Knauf et al., 2002, 2006). In flies, INDY can exchange dicarboxylates for citrate and a proton during an electro-neutral and Na^+^-independent processes (Knauf et al., 2006). On the other hand, worm, bacterial and mINDY mediate transport of citrate in exchange for Na^+^ (Pajor, 2014). Stoichiometric analysis of mINDY reveals 11 transmembrane domains with an exchange rate between Na^+^:citrate of 4:1 (Inoue et al., 2003; Pajor, 2014).

Mutational analysis identify specific highly conserved amino acid motifs required for Na^+^ ion binding, which is subsequently essential for citrate binding. Mutations in this region in either of the two sodium-binding domains of SLC13A5 are associated with autosomal-recessive epileptic encephalopathy with seizures in neonates (Thevenon et al., 2014). It has been speculated that such a severe phenotype is most likely due to the inability of mutated SLC13A5 to bind sodium, which is required for transportation of citrate across the plasma membrane (Thevenon et al., 2014). Similarly, the crystal structure of a bacterial INDY homolog demonstrates that a 1:1 interaction between Na^+^ and citrate facilitate binding to conserved amino acids motifs. Binding induces conformational changes that mediate substrate translocation across the membrane (Mancusso et al., 2012).

## INDY Reduction Extends Longevity

INDY reduction in flies and two different worm homologs extends longevity (Rogina et al., 2000; Fei et al., 2003, 2004; Toivonen et al., 2007; Wang et al., 2009; Rogina and Helfand, 2013; Rogers and Rogina, 2014). We have described effects of a P-element or a GFP protein-trap insertion on fly longevity in twelve *Indy^206^* alleles and described a relationship between longevity extension and the degree of *Indy* mRNA reduction (Rogina et al., 2000; Wang et al., 2009; Rogina and Helfand, 2013; Rogers and Rogina, 2014). In several of the *Indy* alleles, the P-element is inserted within the Hoppel element in the first intron of the *Indy* gene, upstream of the putative translation start site. Several other *Indy* alleles have the P-element inserted upstream from the putative transcriptional start site, which reduce *Indy* transcription and also yield longevity extension (Rogina et al., 2000; Wang et al., 2009; Rogina and Helfand, 2013; Rogers and Rogina, 2014). Further investigation with various *Indy* alleles revealed the extent to which *Indy* alleles were capable of extending lifespan was dependent upon the degree of *Indy* mRNA reduction. When *Indy* levels are reduced about 50% as in *Indy^206^* or *Indy^YC0030^* heterozygous flies, life of the flies is maximally extended, by up to 100%. Accordingly, moderate *Indy* reduction has modest beneficial effect on longevity of ∼17%, as seen in *Indy^EY1442^*, *Indy^EY01458^*, and *Indy^EY13297^* heterozygous male flies (Rogina and Helfand, 2013). Interestingly, there appears to be a threshold for *Indy* reduction as dramatic reduction of *Indy* mRNA, as in *Indy^206^/Indy^206^* homozygous flies, reduces beneficial effects on longevity to about 20%, which is thought to induce a state of starvation (Wang et al., 2009). Such longevity extension was observed in multiple genetic backgrounds but not all, stressing the importance of genetic background in longevity studies (Toivonen et al., 2007; Wang et al., 2009; Rogina and Helfand, 2013).

Recent reports extended studies on the effects of *Indy* reduction to natural populations (Zhu et al., 2014). The authors found that natural population of flies heterozygous for insertion of the Hoppel element experience beneficial effects on fly reproduction and longevity (Zhu et al., 2014). This is consistent with data showing that under standard laboratory conditions heterozygous *Indy^206^* and *Indy^302^* flies laid more eggs during their life compared to control (Marden et al., 2003). Furthermore, INDY reduction does not affect maximal flight velocity, negative geotaxis or resting metabolic rate in heterozygous *Indy^206^* and *Indy^302^* flies (Marden et al., 2003). Together these data highlight the varied and diverse benefits associated with INDY reduction.

## Reduced INDY Mimics Calorie Restriction

Many phenotypes associated with *Indy* reduction are reminiscent of physiological changes induced by CR, which range from starvation sensitivity to changes in fecundity (Bross et al., 2005; Guarente, 2008; Wang et al., 2009; Birkenfeld et al., 2011; Rogers and Rogina, 2014). In that likeness, it has been shown that caloric content of fly diet affects *Indy* mRNA levels showing reduced *Indy* during CR and no further longevity extension when *Indy* mutant flies are on CR (Wang et al., 2009; Rogers and Rogina, 2014). Similar to findings in flies with reduced INDY, *mIndy^–/–^* mice have increased hepatic mitochondrial biogenesis, increased insulin sensitivity, and are protected from the adiposity when kept on high fat diet. Whole-genome microarray analysis comparing *mIndy^–/–^* and *mIndy^–/+^* revealed that 80% of the differences that were observed in these mice were not only related to transcriptional regulatory pathways but also strikingly similar to differences previously described in CR and *ad libitum*-fed mice (Birkenfeld et al., 2011).

Further support that links INDY reduction and CR-mediated longevity is related to the nutrient sensing insulin-signaling pathway. Under standard conditions, insulin signaling is active and allows for downstream activation of FoxO via phosphorylation. When nutrients are scarce, as in CR, insulin signaling is down regulated, which prevents FoxO phosphorylation and allows for nuclear translocation. Similar to CR, flies with INDY reduction have predominantly nuclear FoxO localization (Wang et al., 2009). Moreover, *Indy* mutants show reduced levels of *Drosophila* insulin-like peptides (Dilps) dilp2, dilp3, and dilp5 when kept on high caloric diet (HCD), with levels similar to genetic control flies on a CR diet (Wang et al., 2009). Furthermore, *mIndy^–/–^* mice have increased insulin sensitivity, and are protected from adiposity when kept on high fat diet, which supports a conserved role for INDY in metabolic regulation. The finding that INDY may be interacting with insulin signaling links INDY to a key signaling pathway known to influence aging and metabolic disorders in a variety of species (Kenyon, 2010). Nevertheless, additional studies are merited to connect insulin signaling to longevity that is observed in flies with *Indy* reduction.

## The Role of INDY in Metabolism

INDY functions as a citrate transporter during intermediary metabolism, thus its transport activity directly influences downstream events related to citrate metabolism. When citrate levels are high, glycolysis and fatty-acid β-oxidation pathways are down regulated and fatty-acid synthesis pathway is active. Cytoplasmic citrate is cleaved to oxaloacetate and acetyl-CoA by ATP-citrate lyase. Acetyl-CoA can be used for the biosynthesis of fatty acids, triglycerides, low-density lipoproteins and cholesterol. Additionally, cytoplasmic oxalacetate can be converted to malate and transported by the malate transporter to the mitochondria and used for energy production in the Krebs cycle. Cytoplasmic citrate levels are also regulated by the mitochondrial citrate carrier (CIC, SLC25A1), which transports citrate from the mitochondria thereby contributing to cytoplasmic citrate regulation (Gnoni et al., 2009).

**FIGURE 1:**
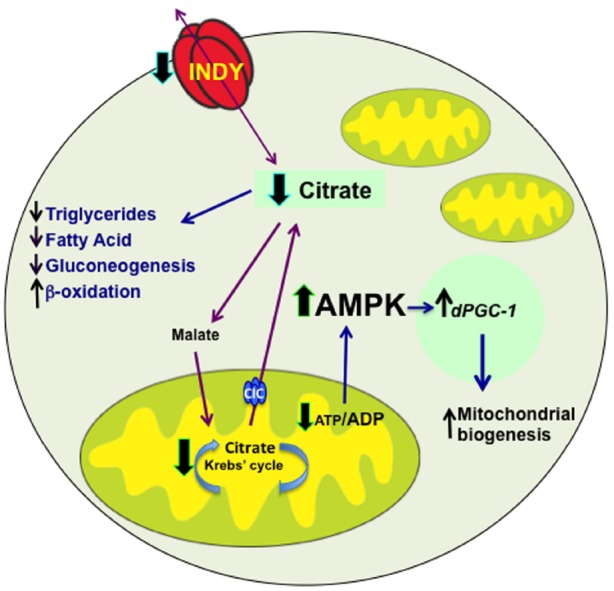
**INDY transport activity affects metabolism.** INDY reduction decreases cytoplasmic citrate levels, which results in decreased synthesis of triglycerides, fatty acids and reduced gluconeogenesis, but increased β -oxidation. Citrate is converted to malate and transported to mitochondria, where when broken down to oxaloacetate, can enter Krebs’ cycle. The Citrate Carrier (CIC, SLC25A1), which is located on the inner mitochondrial membrane transport mitochondrial citrate to cytoplasm. Reduced levels of INDY decrease production of ATP, which activate AMPK. Activated AMPK promotes mitochondrial biogenesis by increasing *dPGC-1* transcription. Blue arrows indicate downstream effects; whereas, black arrows represent changes in expression, red arrows indicate transport of citrate and malate.

Reduced INDY activity in flies and mice alters availability of these substrates during intermediary metabolic processes by reducing citrate transport, subsequently causing reduction of ATP levels (Birkenfeld et al., 2011). Augmenting the ATP/ADP ratio activates AMPK, which, by activating mitochondrial transcriptional co-activator *spargel/dPGC-1*, increases mitochondrial biogenesis to maintain cell energetic requirements (Figure 1; Gershman et al., 2007; Guarente, 2008; Neretti et al., 2009; Birkenfeld et al., 2011; Rogers and Rogina, 2014). This is supported by the presence of increased *PGC-1* mRNA levels and mitochondrial biogenesis in *mIndy^–/–^* mice and the midgut of flies with reduced INDY levels (Birkenfeld et al., 2011; Rogers and Rogina, 2014). Consistent with the role of activated AMPK in insulin sensitivity and lipid metabolism, *mIndy^–/–^* mice have higher lipid oxidation, reduced lipogenesis and increased insulin sensitivity (Birkenfeld et al., 2011).

Environmental manipulations can mimic aging, such as exposure to HCD or paraquat. It was recently shown that such manipulations also increase levels of *Indy* mRNA in the fly midgut, while CR had the opposite effect (Rogers and Rogina, 2014). Given that INDY transports metabolic intermediates, this finding likely represents a response to higher energetic demands for tissue repair during aging or paraquat treatment so that necessary metabolites can more readily reach the TCA or mitochondria for processing. Further evidence that nutrient availability affects *Indy* gene expression has recently been provided showing altered levels of *mIndy* gene expression in rat hepatocytes following glucagon release during early starvation (Neuschafer-Rube et al., 2014). Glucagon binds to the cAMP-dependent and cAMP-responsive element-binding protein (CREB)-binding site in the promoter region of *mIndy* (Neuschafer-Rube et al., 2014). Overnight fasting induces *Indy* mRNA expression, while prolonged starvation decreases expression levels. This is most likely due to the short half-life of glucagon and the subsequent activation of downstream regulatory feedback loops (Neuschafer-Rube et al., 2014). These findings are consistent with decreased levels of *Indy* mRNA found in the livers of mice after 36 h of starvation. Furthermore, increased INDY expression was found in the rat liver after force-feeding large amounts of olive oil, thus supporting a regulatory role for INDY during metabolic activity (Martinez-Beamonte et al., 2011).

## *INDY* Reduction Preserves Intestinal Stem Cell Homeostasis

Aging is a complex process characterized by a loss of homeostasis (Biteau et al., 2010). Preservation of intestinal stem cell (ISC) homeostasis has become an important factor in extending longevity due to a central role for ISCs in preserving normal midgut functions such as food absorption and protection from microorganisms and toxins. Environmental and genetic manipulations that preserve ISC homeostasis promote healthy aging and extend longevity in flies (Biteau et al., 2010). ISC proliferation is significantly increased in the aged intestine due to age-related ROS accumulation, chronic stress, or injuries, which trigger signaling pathways responsible for initiating cell division. However, in the aging midgut, ISCs differentiate at a slower rate, generating non-functional cell populations that are unable to replace damaged midgut cells.

Recently we described a role for INDY in the midgut during intestinal regeneration. INDY is robustly expressed in midgut tissue. Reduce *Indy* levels are associated with dramatic extension of lifespan accompanied by enhanced metabolic activity. We have shown that reduction in *Indy* mediates changes in intermediary metabolism that preserves ISC regenerative homeostasis and intestinal integrity by modulating *dPGC-1* activity (Rogers and Rogina, 2014). Flies with reduced *Indy* mRNA levels have significantly higher *dPGC-1* mRNA levels in the midgut throughout lifespan compared to genetic controls, which exhibit an age-related reduction in the levels of *dPGC-1*. Not only do these conditions promote oxidative stress resistance, but also preserve the redox environment of ISCs, which subsequently preserves proliferative homeostasis and intestinal integrity. Without functional *dPGC-1*, *Indy* mutant flies do not show beneficial changes in mitochondrial physiology, ROS production or intestinal integrity, suggesting that *dPGC-1* mediates some of the regulatory effects of *Indy*-mediated healthspan.

The relationship between *dPGC-1* and *Indy* also has a large impact on *Drosophila* longevity. We showed that *Indy* and *dPGC-1* longevity pathways overlap, which was supported by the observation that flies hypomorphic for *Indy* and *dPGC-1* have a lifespan similar to controls (Rogers and Rogina, 2014). Such findings confirm that *dPGC-1* must be present and functional in order to confer lifespan extension in *Indy* alleles. This is consistent with recent work that has revealed a role for the *dPGC-1* in ISC homeostasis, establishing a connection between mitochondrial function and tissue homeostasis (Rera et al., 2011; Rogers and Rogina, 2012). Our work has extended this finding by suggesting INDY as an upstream regulator of *dPGC-1* activity (Rogers and Rogina, 2014). We suggest that reduced *Indy* increases *dPGC-1* activity, which promotes mitochondrial biogenesis and changes the redox environment of the *Indy* mutant midgut. Such changes preserve tissue homeostasis and ultimately mediate lifespan extension.

## Summary

Longevity studies in worms and flies demonstrated that reduction in *Indy* gene activity is comparable to CR and associated with a longer and healthier life by affecting energy production. Similarly, knockdown of *mIndy^–/–^* in mice mimics CR by altering metabolic activity in the liver (Birkenfeld et al., 2011). The recent work completed by our lab and others support a role for INDY as a regulator of metabolism whose transcriptional levels change in response to calorie content of the food, as well as in response to energetic requirements of the organism (Willmes and Birkenfeld, 2013; Rogers and Rogina, 2014). The similar effects of INDY reduction on metabolism in flies, worms, and mice suggest an evolutionary conserved and universal role of INDY in metabolism. Together, these findings suggest that INDY could be potentially used as a drug target for treatment of obesity and Type II Diabetes in humans. Further investigation on the mechanism of INDY reduction could provide valuable information regarding the means to a healthier and more productive life.

### Conflict of Interest Statement

The authors declare that the research was conducted in the absence of any commercial or financial relationships that could be construed as a potential conflict of interest.
